# A Voluntary Insurance Bill

**Published:** 1914-11-14

**Authors:** 


### NATIONAL INSURANCE ACT DEVELOPMENTS.

### A Voluntary Insurance Bill.

From time to time suggestions have been made for the alteration of the National Insurance Acts so that their compulsory character should be superseded and the insurance be made voluntary. Mr. Bonar Law announced some while ago that if the Opposition came into power they would appoint a committee to consider the question of an alteration in the fundamental basis of the Acts. No definite pledge was given, however, that a system of voluntary insurance would be instituted, although he is reported to have stated that he was satisfied that a voluntary basis would be perfectly feasible. Seeing that the National Insurance Acts have been based upon the idea of compulsion, and that all the machinery has been set up to give effect to that compulsion, it is hardly to be wondered at that those who are connected with the administration of the Acts hold that it would be impossible to make a change to a voluntary system. It is argued that the vested interests created by the Acts would preclude the possibility of the change, no matter how much the relief that might be afforded in certain quarters might be urged in its favour. Quite apart from this difficulty of vested interests, it has been urged that without compulsion State-aided thrift would make progress but little, if any, better than that which was secured by the unaided thrift of those who formerly constituted the friendly societies. If it be premised that insurance is in itself the best way of meeting the requirements of the wage-earning and industrial section of the community in times of sickness and unemployment, so that they shall not have recourse to the Poor Law or to charity for relief, then it is clear that the question as between a compulsory or voluntary system must be decided according to which plan would ensure the greatest number of insured persons without inflicting undue hardship on any considerable section.

### The Voluntary Insurance Bill.

A chance for comparing the two systems is afforded by a Voluntary Insurance Bill,* which was introduced into Parliament towards the close of last session by Sir Richard Cooper, and the problem can in consequence now be examined in some detail. It must, of course, have been quite clear to the introducer that such a Bill could not possibly find its way to the Statute Book on the introduction of a private member. Yet the Bill will have served a useful purpose if it forms the basis for the fuller consideration of the subject. Unfortunately the business of the session did not permit the Bill to be discussed, and, apart from casual notice in the Press, it does not appear to have received any detailed consideration. It is all the more necessary, therefore, to call attention to its provisions.

Covering both Health Insurance and Insurance against Unemployment, the Bill shows evidences of careful preparation, so that it will be seen that a serious attempt has been made to incorporate in the Bill all the necessary details for the complete application of the altered system.

### The Scheme in Outline.

Dealing with the provisions of the Bill in regard to Health Insurance, it is proposed that all British subjects of the age of sixteen and upwards, whether employers or employed, so long as they are wholly or mainly dependent upon their earnings in an employment or regular occupation, shall be entitled to be insured, provided their income does not exceed £160 per annum. There is to be no compulsion, and in consequence there are no provisions, as in the National Insurance Acts, as to voluntary contributors, excepted occupations, or exempted persons. Therefore, the plan of the Bill is undoubtedly more readily applied than that of the present Acts, which has necessitated numberless adjustments to meet the vastly differing requirements of the many different classes of insured persons who have been compulsorily insured.

An option is given to each insured person under the scheme to pay a weekly contribution of either 4d. or 3d. This applies equally to men as to women, and, in fact, there does not appear to be any distinction between the rates of contribution or benefits applicable to the two sexes. In certain circumstances, where, for instance, the wages of an insured person over twenty-one years of age are less than 21s. a week, there are to be reduced rates of contribution, and in the extreme case where wages are less than 14s. a week no contribution is to be payable. This differentiation is similar in principle to that which is already in existence. Apart from this similarity the proposals as to the contributions are very different, for there is to be no direct contribution from the employer, except, as will presently be stated, in the form of a special Excise duty payable annually or at other agreed intervals on the number of persons employed. The employer is, in fact, not to be concerned whether his employees are insured or not. Contribution cards to be stamped by the employer would thus be discarded, and the insured persons, who would have to become members of approved societies in order to obtain the benefits of the insurance, would themselves be responsible for the payment of their own contributions to their societies. In this it will at once be seen that there is a considerable divergence from the existing system. It is not only the liability to insurance that is made optional, but also the rate of contribution, and, perhaps more important still, there is to be no inducement other than the penalties that must necessarily attach to arrears of contribution to ensure that the contributions, when once they have been commenced, will be maintained. It is true, no doubt, that a certain number of employers resent the necessity for attaching the insurance stamps to insurance cards, and would welcome the relief that is proposed by

* Bill 314. Government Publishers. 9d.

this measure, but it must be borne in mind that the comparatively slight inconvenience to the employer in this respect is in many cases of great advantage to the insured person, who would otherwise be tempted to allow his contributions to fall into arrear.

### Administration of Benefits.

The benefits to be derived from the insurance under this proposed scheme would depend upon the rates of contribution paid. Thus a person paying 4d. a week would ordinarily receive the benefits provided under the National Insurance Acts for male employed contributors, while persons contributing 3d. a week would be entitled ordinarily to those of female employed contributors. There is, however, one exception from the list of benefits in that it is proposed that sanatorium benefit would more properly and adequately be dealt with by public health authorities, apart entirely from insurance.

All the benefits except medical benefit are to be administered by approved societies, and the class of deposit contributors is to be abolished. Every approved society would be required to give the benefits specified in the Bill, but it would be possible for a society to increase, reduce, or vary those benefits in accordance with a scheme, provided this were approved by the Insurance Commissioners. Medical benefit, of a similar character to that already provided, would be administered by local medical councils, and the existing Insurance Committees would be abolished. Approved societies would not be directly represented on these medical councils, and thus the administration of medical benefit would pass entirely into the hands of the medical profession. It would, however, be necessary for the councils to enter into arrangements with the societies having members resident in their districts for the provision of the funds required. Failing such agreement, the funds would be provided by the Insurance Commissioners and deducted from the allowances made to the societies in question. It would also be competent for the Insurance Commissioners to agree to other arrangements being made for the administration of medical benefit when the arrangements made by the local medical councils are unsatisfactory. In spite of the proposal that the administration should pass entirely into the hands of the medical profession, the condition that agreements would have to be made between the councils and individual societies would involve many problems not agreeable to the profession.

### The Finance of the Scheme.

Turning to the financial side of the proposals, it has already been pointed out that there are no longer to be contributions payable by the employers. To take the place of these contributions it is proposed that each employer should pay an annual licence duty in respect of each employee. When the employment is for the purpose of the employer's trade or business, and the trade is not one to which the unemployment provisions of the Bill would apply, the licence duty is at the rate of 5s. a year. This is a lower sum than that at present paid in the form of employer's contribution, and it is apparently the intention of the framer of the measure to afford some relief to employers by this means. When persons are not employed for the purposes of the employer's trade or business—for example, in the case of domestic servants—the duty is to be at the rate of 5s. where one such person only is employed, 10s. each where two are employed, and 15s. each where three or more are employed. This last figure is somewhat higher than the present contribution, and is apparently designed as a tax upon luxury. Various slight modifications are made to allow for casual labour and for periods of service less than one year. The licence duties in these cases are at a slightly higher rate. It is also to be noted that the rate of duty to be paid in the case of an alien employee is double the ordinary rate, although the employee in question is not entitled to be insured unless already insured under the existing Acts.

These special licence duties would go to form a fund which would be directly applicable for the purposes of insurance. They would, however, require to be supplemented, possibly to a large extent, from the Consolidated Fund derived from the general taxation of the country. Here, again, there is a fundamental difference between the proposals and the existing scheme, for instead of the Government contribution taking the form of an addition to the benefits it is to be given directly as an addition, at yearly or other intervals, to the contributions that have been paid to the societies. The State contribution is to be 3d. for each weekly contribution of 3d. or 4d. from the members themselves, so that the societies obtain a smaller proportional subsidy for the larger contribution. This might have the effect of reducing the contributions from insured persons all the way round to 3d., more particularly as the opportunity for extra independent provision is allowed. If this were the result the charge to the State would be heavy, and the inducement to persons entitled to become insured to join an approved society would be ample.

### Valuations not Required.

There is one feature of the finance of the present system that is conspicuous by its absence—namely, that no provision appears to be made for the actuarial valuations of the societies, and no means of adjusting the rates of benefit to the resources derived from the contributions. It would seem that in this respect the State would be called upon to ensure the solvency of the societies. Direct provision is made to this effect so far as associate members are concerned—that is, for those members who are taken over from the deposit contributor class—and maladministration of benefits is to be penalised. This lack of provision for actuarial valuations is one of the weakest features of the Bill, for it means that the liabilities of the Government would be at all times an unknown quantity.

